# Aberrant DNA methylation of imprinted loci in hepatocellular carcinoma and after *in vitro* exposure to common risk factors

**DOI:** 10.1186/s13148-015-0053-9

**Published:** 2015-02-27

**Authors:** Marie-Pierre Lambert, Pierre-Benoit Ancey, Davide Degli Esposti, Marie-Pierre Cros, Athena Sklias, Jean-Yves Scoazec, David Durantel, Hector Hernandez-Vargas, Zdenko Herceg

**Affiliations:** Epigenetics Group, International Agency for Research on Cancer (IARC), 150 Cours Albert-Thomas, 69008 Lyon, France; Current address: Epissage alternatif et progression tumorale, Centre de Recherche en Cancérologie de Lyon (CRCL), 28 rue Laennec, 69008 Lyon, France; Institut Gustave Roussy, 114 Rue Edouard Vaillant, 94805 Villejuif, France; INSERM U871, Molecular physiopathology and new treatments of viral hepatitis, Centre de recherche en cancérologie (CRCL), 151 Cours Albert-Thomas, 69008 Lyon, France

**Keywords:** Methylome, Promoter methylation, Imprinting, Liver cancer

## Abstract

**Background:**

Hepatocellular carcinoma (HCC) is among the most frequent human malignancies and a major cause of cancer-related death worldwide. It is characterized by late detection and fast progression, and it is believed that epigenetic disruption may be one of the molecular mechanisms leading to hepatocarcinogenesis. Previous studies from our group revealed that HCC tumors exhibit specific DNA methylation signatures associated with major risk factors and tumor progression. Imprinted genes are mono-allelically expressed in a parent-of-origin-dependent manner and have been suggested to be more susceptible to deregulation in cancer. To test this notion, we performed a targeted analysis of DNA methylation in known imprinted genes, using HCC samples and *in vitro* models of carcinogenic exposure.

**Results:**

Analysis of HCC DNA methylation in two independent datasets showed that differentially methylated loci are significantly enriched in imprinted genes. Most of the promoters of imprinted genes were found hypomethylated in HCC tumors compared to surrounding tissues, contrasting with the frequent promoter hypermethylation observed in tumors. We next investigated the status of methylation of the imprinting control region (ICR) of different imprinted clusters and found that the 15q11-13 ICR was significantly hypomethylated in tumors relative to their surrounding tissues. In addition, expression of imprinted genes within this cluster was frequently deregulated in a gene-specific manner, suggesting distinct mechanisms of regulation in this region. Finally, primary human hepatocytes and hepatocyte-like HepaRG cells displayed higher methylation variability in certain imprinted loci after natural hepatitis B virus (HBV) infection and after lipid accumulation, respectively.

**Conclusion:**

The methylation status of a large panel of imprinted genes was found deregulated in HCC, suggesting a major role of this mechanism during hepatocarcinogenesis. *In vitro* models support the hypothesis of imprinted gene methylation as a potential marker of environmental exposures.

**Electronic supplementary material:**

The online version of this article (doi:10.1186/s13148-015-0053-9) contains supplementary material, which is available to authorized users.

## Background

Epigenetic mechanisms are believed to play a major role in gene regulation during development and differentiation as well as disease. Indeed, deregulation of gene expression due to epigenetic events has been reported to play a role in carcinogenesis. The relative importance of epigenetics in defining the mammalian transcriptome in normal and disease states is still unknown. However, the mammalian genome contains a small number of genes for which epigenetic gene regulation has been shown to play a major role in transcriptional control, called imprinted genes. Genomic imprinting, which results in the parental-specific expression of specific genes, plays an important role in normal growth and development [1]. To date, between 100 and 200 imprinted genes have been described in mammals, many of them being well conserved between mouse and human (http://www.har.mrc.ac.uk/research/genomic_imprinting/, http://igc.otago.ac.nz/home.html). Disruption of imprinting can result in a number of human pathologies, some of which predispose to cancer [2]. Indeed, genomic imprinting subjects mammals to a greater genomic risk because a mutation in one allele (either genetic or epigenetic) can result in the absence of one or more gene products, thus leading to a number of well-known imprinting disorders, including Beckwith-Wiedemann, Silver-Russell, Prader-Willi, and Angelman syndromes [3-5]. In cancer, loss of imprinting has been largely reported, insulin-like growth factor 2 (IGF2) being the most studied locus [6-8]. However, little is known about the global control of imprinting regions in specific types of human cancer.

Here, we were interested in the potential role of imprinting deregulation in hepatocellular carcinoma (HCC). HCC represents an endemic burden worldwide, partially due to delayed diagnosis and multiple risk factors that contribute to a permanent high incidence [9,10], stressing the need to characterize potential new early biomarkers. Together with other liver diseases, HCC represents a complex pathology due to its heterogeneity in origin and outcome. Well-known risk factors include chronic hepatitis B virus (HBV) and hepatitis C virus (HCV) infection, steatohepatitis, toxic, metabolic, and immune-related conditions [11]. Common to most of these risk factors is the establishment of proliferative disease, frequently followed by malignancy [12].

In the liver, the sequential progression to carcinoma has been linked to changes at the genetic and epigenetic level, including aberrant induction of pathways related to proliferation and development, and inactivation of tumor suppressor genes [13,14]. Few studies have already reported disruption of imprinted genes in HCC. Among them, the *IGF2/H19* cluster has been the most studied [15-17]. In a similar way, delta-like 1 homolog (*Drosophila*) (*DLK1*), a paternally expressed gene, has been found to be upregulated in HCC tumors [18]. Deregulation of *IGF2*, *H19*, and *DLK1* has been shown to be associated with methylation changes and leading to cell proliferation promotion while its interference triggers inhibition of cell growth, colony formation, and tumorigenicity in HCC cell lines [18], suggesting that the deregulation of imprinted genes may promote cancer development.

Although the role of imprinted genes in carcinogenesis is accepted, there are currently few data on their regulation in tumors. Thus, we investigated the methylation status of a large panel of imprinted genes in HCC tumors and surrounding tissues using two independent datasets. Using *in vitro* models of HCC risk factors, we studied methylation variability in selected imprinted loci.

## Results

### DNA methylation profiling reveals enrichment at imprinted loci in HCC

**Figure 1:**
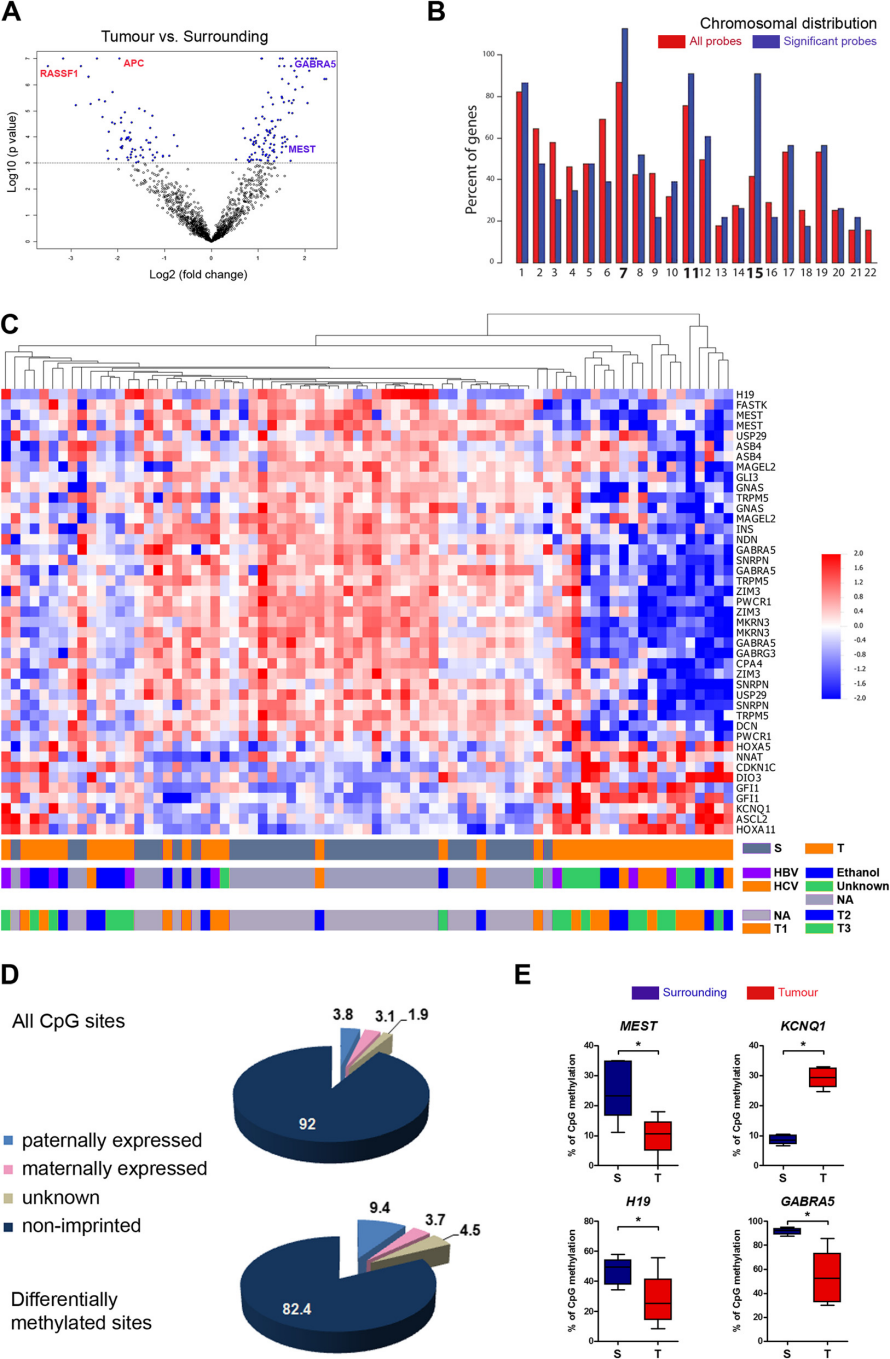
**Imprinted genes are differentially methylated in HCC. (A)** Volcano plot for the comparison of HCC tumors *vs.* adjacent tissues (surrounding/tumor). The horizontal line defines the *P* value threshold of 0.001. Two representative hypermethylated (*APC* and *RASSF1*) and hypomethylated (*GABRA5* and *MEST*) genes are shown respectively in red and blue fonts. **(B)** Distribution of differentially methylated CpG sites (*n* = 244) according to chromosomal location (blue bars). Reference proportion for all probes is shown as red bars. **(C)** Heatmap of differentially methylated imprinted CpG sites (*n* = 43), with high methylation represented in red and low methylation in blue. Annotations in the lower bars correspond to tumor *vs.* surrounding tissues, associated risk factors, and tumor stage. No data is shown for surrounding tissues (NA). **(D)** Estimated proportion of imprinted genes in the total dataset (upper pie chart) and the differentially methylated CpG sites (lower pie chart). Preferential maternal and paternal expressions are shown separately. **(E)** Validation of selected imprinted genes was assessed by pyrosequencing using 5 tumor/surrounding pairs. *P value <0.05.

Our previous DNA methylation bead array analysis revealed a strong panel of genes differentially methylated in 38 HCC tumors compared with their matched surrounding tissues [19], including genes already known as deregulated in HCC like *RASSF1A* or *APC* [20,21] (Figure 1A). Interestingly, this 244-CpG signature (corresponding to 184 genes) comprises also other genes not reported as deregulated in HCC. Thus, further analyses of differentially methylated genes in HCC were performed in order to better characterize the deregulation associated with those epigenetic changes. A chromosomal location analysis of these genes revealed an enrichment in specific chromosomes 7, 11, and 15 (Figure 1B). Indeed, 28% of differentially methylated positions (DMPs) were comprised in those three chromosomes. As chromosomes 7, 11, and 15 have been all reported to contain clusters of imprinted genes, we decided to test the overlap of our HCC signature with a comprehensive list of 228 predicted or established human imprinted genes (http://www.geneimprint.org/). Out of a total of 813 genes analyzed (corresponding to 1,505 CpG sites), 59 imprinted genes (corresponding to 153 CpG sites) were present in the reference list. In this sense, the GoldenGate bead arrays represent a biased selection of cancer-related loci, including imprinted genes (8.8% of all genes in the array consist of imprinted genes, in contrast to approximately 1% described in the human genome). Interestingly, an unsupervised clustering analysis performed using only the 153 imprinted CpG sites revealed that they are able to discriminate tumor samples from surrounding tissues (Additional file 1: Figure S1). When considering the differentially methylated genes in the HCC tumor *vs.* surrounding comparison, we found 27 imprinted genes out of 184 total significant genes, more than expected by chance (*P* = 0.0001, two-tailed Fisher’s exact test). This significant enrichment was also observed at the CpG site level, with 43 CpG sites in imprinted loci out of a total of 244 sites (*P* = 0.0002, two-tailed Fisher’s exact test). Differential methylation of imprinted genes in HCC was found to be independent of tumor grade, stage, and risk factor exposures (Figure 1C). In total, 28% of the imprinted CpG sites analyzed by the array were found significantly differentially methylated in HCC, relative to surrounding tissues. Interestingly, most of them (79%) were found hypomethylated in HCC tumors compared with surroundings, including *H19*, *MEST*, and *GABRA5*, contrasting with the common phenomenon of promoter hypermethylation observed in tumors (Figure 1C). *GABRA5* promoter showed the highest fold change reduction in methylation in tumors relative to adjacent tissues (5.16-fold change) (Table 1). Within the 9 imprinted CpG sites hypermethylated in tumors, 5 were maternally expressed (*KCNQ1*, *CDKN1C*, *HOXA5*, *HOXA11*, *ASCL2*) (Figure 1C, Table 1). The original dataset includes 8.8% of imprinted genes, maternal and paternal origin being similarly represented (Figure 1D). However, when discriminating imprinted genes by their maternal or paternal contribution, we found that most of the enrichment was due to differential methylation of paternally expressed genes, with a representation of 3.8% of the total genes in the bead array compared to 9.4% in the list of differentially methylated genes (Figure 1D). Indeed, among the 43 significant imprinted loci, 9 are known to be associated with maternally expressed and 23 with paternally expressed genes (Table 1), suggesting that the parental origin of the gene may be related to its susceptibility to epigenetic disruption in carcinogenesis.

**Table 1:** **Imprinted genes differentially methylated in HCC tumors compared to surrounding tissues**

**Unique ID (CpG site)**	***P*** **value**	**FDR**	**Fold change**	**Symbol**	**Expressed allele**
USP29_E274_F	<1e-07	<1e-07	0.29	*USP29*	Unknown
MKRN3_P108_F	2E-07	5E-05	0.25	*MKRN3*	Paternal
ZIM3_E203_F	2E-07	5E-05	0.24	*ZIM3*	Unknown
GABRA5_E44_R	4E-07	7E-05	0.28	*GABRA5*	Paternal
ZIM3_P718_R	4E-07	7E-05	0.20	*ZIM3*	Unknown
MKRN3_E144_F	8E-07	0.0001	0.22	*MKRN3*	Paternal
PWCR1_E81_R	1E-06	0.0001	0.45	*PWCR1*	Paternal
GABRA5_P862_R	1E-06	0.0001	0.19	*GABRA5*	Paternal
GABRG3_E123_R	2E-06	0.0002	0.21	*GABRG3*	Paternal
TRPM5_E87_F	5E-06	0.0003	0.29	*TRPM5*	Paternal
TRPM5_P979_F	7E-06	0.0004	0.36	*TRPM5*	Paternal
GABRA5_P1016_F	1E-05	0.0006	0.34	*GABRA5*	Paternal
MEST_P62_R	2E-05	0.0009	0.32	*MEST*	Paternal
ZIM3_P451_R	4E-05	0.0014	0.51	*ZIM3*	Unknown
TRPM5_P721_F	1E-04	0.0023	0.23	*TRPM5*	Paternal
SNRPN_P230_R	1E-04	0.0024	0.50	*SNRPN*	Paternal
INS_P804_R	1E-04	0.0027	0.31	*INS*	Paternal
GLI3_E148_R	3E-04	0.0043	0.55	*GLI3*	Maternal
CPA4_P1265_R	3E-04	0.0048	0.41	*CPA4*	Maternal
GFI1_E136_F	4E-04	0.0059	4.35	*GFI1*	Paternal
PWCR1_P811_F	6E-04	0.008	0.50	*PWCR1*	Paternal
CDKN1C_P626_F	0.001	0.0109	2.17	*CDKN1C*	Maternal
ASCL2_P360_F	0.001	0.0111	4.35	*ASCL2*	Maternal
HOXA11_P698_F	0.001	0.0111	3.70	*HOXA11*	Maternal
DCN_P1320_R	0.001	0.0115	0.33	*DCN*	Unknown
NDN_P1110_F	0.001	0.0126	0.39	*NDN*	Paternal
FASTK_P598_R	0.001	0.0127	0.54	*FASTK*	Maternal
MAGEL2_P170_R	0.002	0.0141	0.40	*MAGEL2*	Paternal
GNAS_P86_F	0.002	0.0148	0.53	*GNAS*	Isoform dependent
HOXA5_E187_F	0.002	0.0157	2.44	*HOXA5*	Maternal
MEST_P4_F	0.002	0.016	0.33	*MEST*	Paternal
DIO3_P674_F	0.002	0.0167	3.23	*DIO3*	Unknown
GNAS_E58_F	0.002	0.0167	0.40	*GNAS*	Isoform dependent
ASB4_P52_R	0.002	0.017	0.37	*ASB4*	Unknown
NNAT_P544_R	0.002	0.0175	1.41	*NNAT*	Paternal
MAGEL2_E166_R	0.003	0.0187	0.41	*MAGEL2*	Paternal
SNRPN_E14_F	0.003	0.0188	0.49	*SNRPN*	Paternal
KCNQ1_P546_R	0.003	0.0198	1.85	*KCNQ1*	Maternal
USP29_P205_R	0.004	0.0252	0.53	*USP29*	Unknown
SNRPN_seq_18_S99_F	0.004	0.0261	0.50	*SNRPN*	Paternal
GFI1_P208_R	0.004	0.0274	2.44	*GFI1*	Paternal
H19_P541_F	0.005	0.0289	0.46	*H19*	Maternal
ASB4_E89_F	0.005	0.029	0.53	*ASB4*	Unknown

FDR, false discovery rate.

In order to validate this data, we designed specific pyrosequencing assays, allowing a quantitative measurement of DNA methylation on several successive CpG sites. We selected differentially methylated imprinted genes located on different chromosomes and showing opposite methylation profiles between tumors and surrounding tissues (*MEST*, *H19*, and *KCNQ1* as well as *GABRA5* promoters). Their methylation status was assessed in five pairs of HCC samples. The four selected regions displayed differential methylation, *MEST*, *H19*, and *GABRA5* being hypomethylated in tumors compared with adjacent tissues, while *KCNQ1* exhibited hypermethylation of its promoter, confirming the bead array data (Figure 1E).

In summary, differential methylation of imprinted genes is a frequent finding in a series of HCC samples, relative to their matched adjacent tissues. As a group, they tend to be hypomethylated in HCC and their methylation values are able to discriminate between tumors and surrounding tissues. Moreover, paternally imprinted genes are overrepresented in the set of differentially methylated loci, relative to HCC surrounding tissues.

### Specific deregulation of the 15q11-13 ICR in HCC

**Figure 2:**
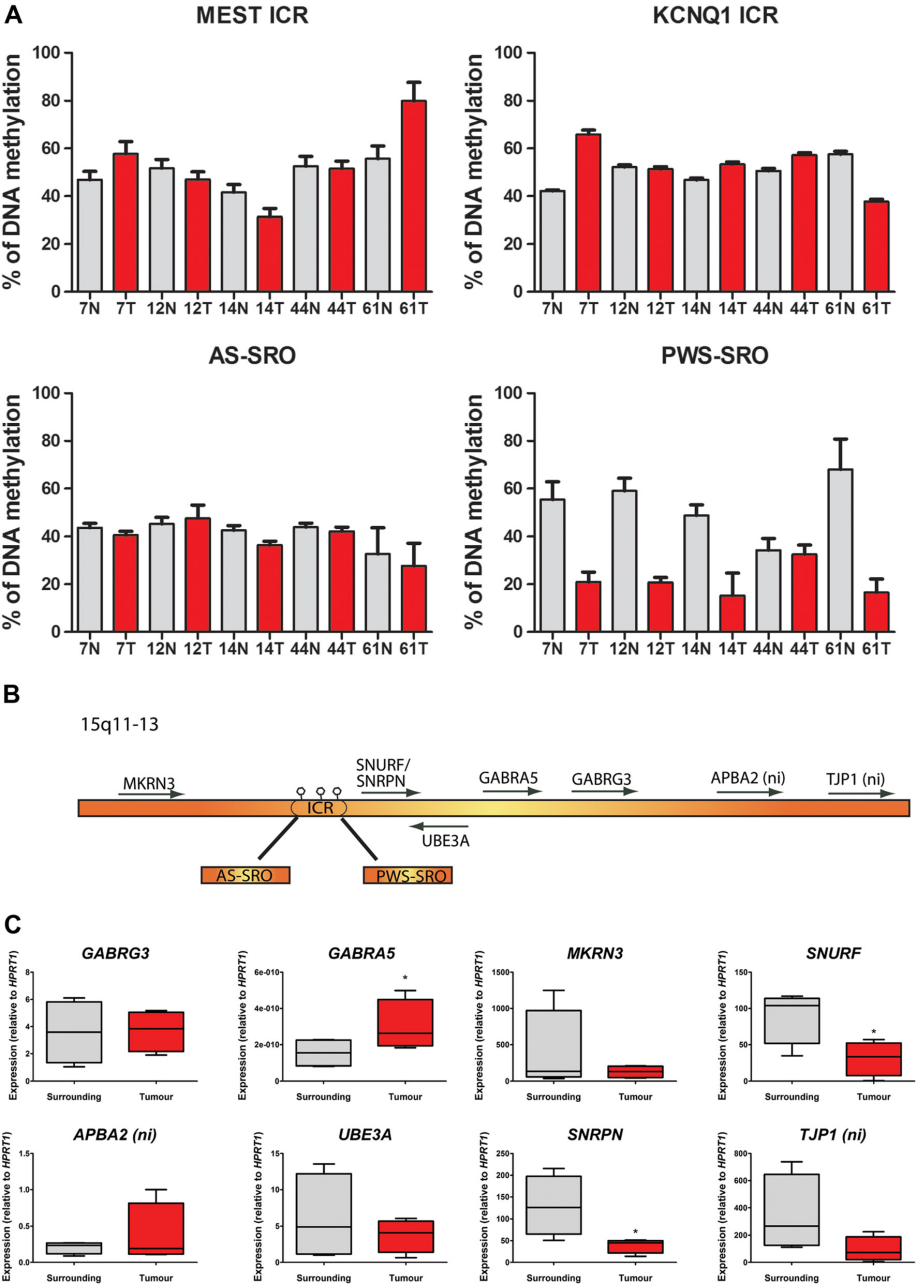
**Deregulation of the 15q11-13 cluster in HCC. (A)** Pyrosequencing assays to assess Tumor *vs.* Surrounding methylation at imprinting control regions (ICR) for *MEST* and *KCNQ1* (n = 5 pairs). The ICR for the 15q11-13 cluster, composed of the Prader-Willi Syndrome region (PWS-SRO) and the Angelman Syndrome region (AS-SRO), was also assessed. **(B)** Diagram of the 15q11-13 cluster showing the different regions of study, including the ICR and two control non-imprinted (ni) genes (*APBA2* and *TJP1*). **(C)** qRT-PCR for selected genes in the 15q11-13 cluster comparing tumors and adjacent tissues (n = 5 tumor/surrounding pairs). *P value <0.05.

The organization of imprinted genes within clusters allows them to share common regulatory elements, such as non-coding RNAs and differentially methylated regions (DMRs). When these regulatory elements control the imprinting of one or more genes, they are known as imprinting control regions (ICRs) and exhibit specific epigenetic features. Due to their key role in imprinting regulation, we examined by pyrosequencing the methylation status of three well characterized ICRs (that were found differentially methylated) in a small panel of HCC tumors and related adjacent tissues (*n* = 5 pairs). The locations of those three ICRs were determined based on previous reports [22-24]. The ICR controlling the 15q11-13 is composed of a sequence adjacent to the *SNRPN* promoter (PWS-SRO) and a sequence located 35 kb upstream (AS-SRO) [24]. While no consistent changes in DNA methylation were observed in *MEST*, *KCNQ1* ICR, and AS-SRO region in HCC tumors and paired surroundings, a significant hypomethylation was detected in HCC in PWS-SRO region (*P* value <0.005), in agreement with the trend of methylation observed at promoter regions (Figure 2A).

As the ICR is a key regulatory element for imprinted genes, we next investigated the impact of the hypomethylation of the PWS-SRO ICR sequence (depicted in Figure 2B) observed in HCC tumors on the expression of the genes comprised in the 15q11-13 cluster. Two genes, *TJP1* and *APBA2*, reported as non-imprinted were included as control. Most of the genes within the cluster were found deregulated in HCC tumors compared with surrounding tissues as assessed by quantitative real-time PCR (qRT-PCR). While most of them were found repressed in tumors (*SNRPN*, *SNURF*, *TJP1*, *MKRN3*), *GABRA5* and *APBA2* were found upregulated in tumors compared with adjacent tissues. No change of expression was observed for *GABRG3* and *UBE3A* (Figure 2C). The loss of methylation observed at the promoter as well as the ICR level is not fully reflected at the transcriptional level within the cluster. Indeed, only *GABRA5* and *APBA2* expression showed an inverse correlation with ICR methylation data.

These data suggest that differential methylation of imprinted genes in HCC is not necessarily linked to differences in methylation in the corresponding ICRs. However, the ICR of one important imprinted locus (15q11-13) displays striking differential methylation in HCC. Contrary to expectation, the hypomethylation observed at the ICR does not necessarily trigger an overexpression of genes within this region suggesting that different regulatory mechanisms may be involved.

#### TCGA data analyses

**Figure 3:**
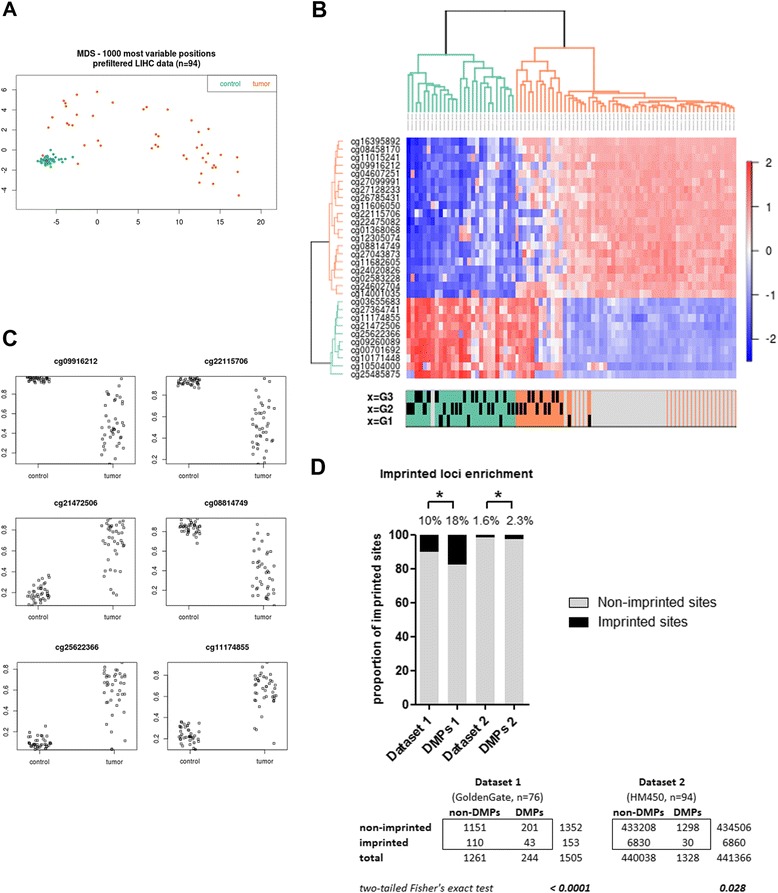
**TCGA replication analysis.** HM450 genome-wide methylation data was downloaded from TCGA (LIHC) for 47 available HCC tumor/surrounding pairs (see Methods). **(A)** Multi-dimensional scaling (MDS) plot showing differential clustering of control *vs.* tumor tissues. **(B)** Differential methylation analysis using paired (tumor/surrounding) linear regression identified 1,328 differentially methylated positions (DMPs); 30 sites (out of 1,328) corresponding to known imprinted loci were used to build an unsupervised cluster. The corresponding heatmap shows the normalized methylation data in a blue-red scale (from lower to higher methylation). Tumor grade is shown in the annotation panel as G1, G2, or G3. **(C)** Example plots of methylation data for 6 of the top differentially methylated imprinted sites in the TCGA HCC tumor *vs.* surrounding comparison (*n* = 47 tumor/surrounding pairs). **(D)** Enrichment analysis comparing the proportion of differentially methylated positions (DMPs) found in imprinted loci for dataset 1 (our original GoldenGate analysis) and dataset 2 (TCGA HM450 analysis). The barplot shows a significant enrichment in imprinted loci in both datasets. The data used to calculate enrichment and the corresponding *P* values (Fisher’s exact test) are shown in the lower panel. *P value <0.05.

The results described above suggest that aberrant methylation of imprinted sites is a frequent finding in HCC tissues, when comparing the methylation profiles to their matched non-tumor tissues. To further validate this finding, we next analyzed the genome-wide methylation data of an independent dataset consisting of 47 HCC samples and their 47 matched surrounding tissues (The Cancer Genome Atlas (TCGA) Data Portal [https://tcga-data.nci.nih.gov/tcga/]). These samples were processed with an alternative platform, the last version of Illumina Infinium arrays (HM450) that interrogates the DNA methylation status of more than 450 K sites across the human genome. Raw data files were downloaded from the TCGA repository and processed as described in Methods. Methylation data was able to discriminate most tumors from their surrounding tissues (Figure 3A). Differential methylation between HCC and surrounding tissues performed at the single site level showed 1,328 DMPs (Additional file 2: Figure S2A).

Next, we studied the enrichment of imprinted loci within those lists. As shown in Figure 3B, C, a subset of 30 imprinted sites (out of 1,328 differentially methylated sites) were differentially methylated with the thresholds used in this analysis (false discovery rate (FDR)-adjusted *P* value <0.05, and change in methylation of at least 40%). Most of these sites were hypomethylated, as was the case for our first dataset (Figure 1 and Additional file 3: Figure S2B). Although HM450 bead arrays have a lower relative representation of imprinted loci when compared to GoldenGate arrays (1.6% *vs.* 10.2% of all sites, respectively), we also observed an enrichment of these features in the list of differentially methylated sites (from 1.6% to 2.3%, *P* value of Fisher’s exact test = 0.028) (Figure 3D). Of note, there was also an increase in the proportion of paternally expressed genes in the list of DMPs, relative to the total imprinted sites contained in the HM450 arrays (from 34% of imprinted sites in HM450 to 46% of the differentially methylated imprinted loci).

Analyses at the regional level identified 160 DMRs when comparing tumor *vs.* surrounding tissues. Of the 160 HCC-related gene DMRs, there were 6 that corresponded to known imprinted genes (that is, *ASCL2*, *ATP10A*, *DLX5*, *GATA3*, *NKX6-2*, and *OTX1*). This is three times more than expected by chance (*P* < 0.014, hypergeometric test).

In summary, the analyses of a second dataset support a significant enrichment of imprinted genes differentially methylated in HCC. This confirms the findings of the first dataset performed in an independent series of samples and a different bead array format.

### Imprinting methylation after *in vitro* risk factor exposure of human hepatocytes

**Figure 4:**
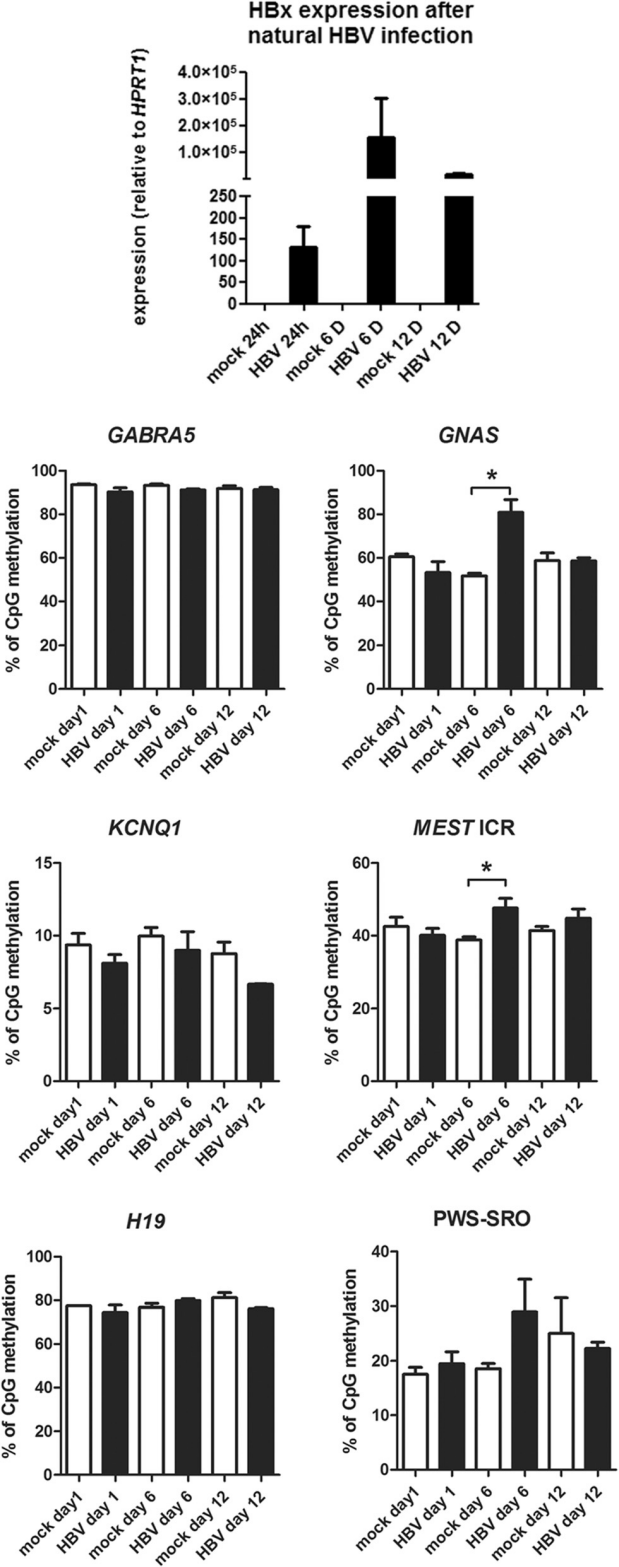
***In vitro*** **models of risk factor exposure.** Primary human hepatocytes were naturally infected with HBV. Efficiency was monitored by qRT-PCR of HBx transcript (upper panel). DNA extracted at different time points was used for pyrosequencing analysis of selected imprinted regions comparing mock to HBV-infected hepatocytes (lower panels).

The results described above suggest that methylation of imprinted loci is frequently lost in HCC. This finding was independent of the risk factor associated to HCC development (that is, HBV, HCV, alcohol). We therefore sought to evaluate variations on DNA methylation levels at imprinted loci using two *in vitro* models: natural HBV infection of primary human hepatocytes (PHHs) and induction of steatosis in hepatocyte-like HepaRG cells. Our assumption was that methylation of imprinted loci could be sensitive to exposure to either risk factor. In the first case, we used PHH infected with HBV during different time points. Infection was evident after 24 h and up to 12 days, as assessed by qRT-PCR expression of HBV X protein coding sequence (HBx) (Figures 3 and 4). DNA extracted from control and HBV-infected conditions was used to quantify methylation in a panel of imprinted or imprinted-related loci. No significant differences were observed after 1 day of HBV infection in any of the selected loci. However, *MEST* and *GNAS* were transiently hypermethylated after 6 days of exposure to HBV infection as assessed by pyrosequencing (Figure 3A).

In a similar way to HBV, we studied another risk factor for HCC using an *in vitro* model of steatosis. We used differentiated HepaRG cells to model steatosis progression, as previously described [25]. HepaRG cells were exposed to increasing concentrations of oleate/palmitate (O/P) to induce lipidic accumulation, as assessed by Nile Red staining (Additional file 3: Figure S3A). Under these conditions, 2 mM O/P was able to significantly increase the methylation of *GNAS* promoter after 2 days of exposure. There were no significant differences in methylation for other imprinted loci (Additional file 3: Figure S3B).

Together, these two *in vitro* models suggest that imprinted loci may be susceptible to differential methylation in response to environmental exposures related to development of HCC.

## Discussion

We have shown here that imprinted genes, as a group, are able to discriminate HCC samples from their adjacent tissues. Indeed, differential methylation in HCC is highly enriched in imprinted genes, especially those that are paternally expressed. In one particular imprinted cluster (15q11-13) differential methylation was extended to the ICR and was associated with deregulated gene expression. We have also shown enrichment on differentially methylated imprinted sites in an independent HCC dataset (TCGA) using a different version of Illumina bead arrays. In addition, using *in vitro* models we show that methylation of some imprinted loci may be more sensitive to variation in response to known HCC risk factors, such as HBV infection and lipidic accumulation.

Imprinting is defined as the parental allele-specific expression of a very limited set of genes. These genes play a key role during embryonic development and adult metabolism, and their expression is tightly regulated allowing correct cell growth and proliferation. Indeed, monoallelic expression of imprinting genes ensures that the levels of proteins for which they encode are regulated. The failure to precisely control their expression may result in developmental abnormalities, as illustrated by a number of hereditary overgrowth or neurological syndromes, including Beckwith-Wiedemann syndrome. This tight regulation depends on epigenetic marks, mainly methylation marks established in a parental specific manner during embryogenesis. In normal somatic cells, the stability of the marked regions is maintained through each cellular replication by at least the DNA methyltransferase DNMT1 during all our life. However, a disruption of imprinting status, so-called loss of imprinting (LOI) is frequently observed in a large number of tumor types [26-30]. The high frequency of LOI as well as its early emergence in tumors [31,32] makes LOI detection an advantageous tool for early diagnosis and detection. In this sense, non-cancer surrounding tissues are known to accumulate DNA methylation changes. However, as we have shown previously [19], the global profile of DNA methylation from surrounding tissues is closer to the profile of non-malignant liver adenoma samples, as compared to HCC samples. This suggests that aberrant DNA methylation progresses in intensity from normal to surrounding and to malignant tissues. Whether a similar pattern is observed for imprinting genes would be an interesting subject for future research. Importantly, the patterns of deregulated methylation at imprinted loci described in our manuscript are independent of the putative etiology of the samples (that is, HBV, HCV, or alcohol consumption).

Few studies have already reported disruption of imprinted genes in HCC [18,33]. Among them, the IGF2/H19 cluster has been the most studied [15-17]. In most normal adult tissues, only the paternal allele of *IGF2* is expressed, whereas only the maternal allele of H19, which is located close to *IGF2*, is expressed. In HCC, this balance of expression is lost. Increased expression of the *IGF2* gene has been reported to be associated with loss of adult-type promoter (P1) transcription, re-imprinting of the fetal-type promoters (P2 to P4), and expression of both alleles of the H19 gene [33-36]. Upregulation of IGF2 and H19 can promote cell proliferation in liver cells. In the same manner, delta-like 1 homolog (*Drosophila*) (*DLK1*), a paternally expressed gene, has been found to be upregulated in HCC tumors. This significant increase of DLK1 expression has been shown to be associated with methylation changes and leading to cell proliferation promotion while its interference triggers inhibition of cell growth, increased colony formation, and tumorigenicity in HCC cell lines [18] suggesting that the deregulation of imprinted genes may promote cancer development.

Here, for the first time, we identified a large panel of imprinted genes altered in HCC. Indeed, a remarkable number of imprinted promoters were found hypomethylated in HCC tumors compared with surrounding tissues, while only few of them that were hypermethylated. Analyses on the specific 15q11-13 region revealed that the changes at the imprinted promoter were correlated with the loss of methylation observed also at the imprinted control region (ICR), suggesting a more global deregulation within this cluster. Maybe contrary to expectation, these changes at the methylation level are not fully reflected at the transcriptional level. Indeed, most of the genes within this cluster were found downregulated in tumors compared to surrounding tissues with the exception of the gene encoding for the GABA type-A receptor alpha5 subunit, *GABRA5*, essential for fast inhibitory neurotransmission and critical in brain function [37]. GABA(A) receptor alpha5 subunit has been associated with autism and bipolar disorder [38,39] but *GABRA5* was never reported as deregulated in cancer. We may hypothesize that the increase of *GABRA5* expression observed can promote cancer development; however, further analyses are needed. Failure to establish a proper imprint of this region in humans has been already described to result in the neurobehavioral disorders, Prader-Willi syndrome (PWS) and Angelman syndrome (AS) [40]; however, this is the first time that an association with cancer is established. In a similar way, we show that the imprinted genes mesoderm specific transcript (*MEST*) and G protein alpha stimulating activity polypeptide 1 complex (*GNAS*) may be sensitive to differential methylation in response to specific risk factors, although further studies will be required to define the kinetics and stability of those changes. In this sense, our *in vitro* systems better reflect the response to acute exposures. Long-term hepatocyte culture systems or animal models would better define the kinetics of imprinting deregulation under specific environmental exposures.

## Conclusions

In summary, we report the HCC-dependent hypomethylation of a large panel of imprinted genes and replicate this finding in an independent dataset. There is a remarkable bias between paternally and maternally imprinted genes within differentially methylated loci. One of the most significantly deregulated loci in HCC corresponds to the region controlled by the 15q11-13 ICR, which includes the maternally imprinted *GABRA5* gene.

## Methods

### Samples

All patients included in the study were referred for treatment to Edouard Herriot Hospital in Lyon, France, between 1997 and 2009, and have been previously described [19]. Thirty-eight patients with HCC were selected for analysis; in all cases, cryopreserved samples from the primary tumor were available for study. In 30 patients, paired cryopreserved samples of adjacent non malignant tissue were also available.

### HBV infection model

HBV inocula were prepared as described [41]. Shortly, HBV was concentrated from the supernatant of HepG2.2.15 cells using centrifugal filter devices and tittered by HBV-DNA dot blot analysis after sedimentation into a CsCl density-gradient to determine enveloped DNA-containing viral particles. PHHs were isolated from surgical liver resections, cultured, and infected with HBV as described [42,43]. Infected PHH and corresponding controls were kept for 1, 6, and 12 days. Supernatants were obtained to validate the efficiency of infection by ELISA, and nucleic acids were extracted for expression and DNA methylation analyses.

### Steatohepatitis model

HepaRG cells (6 × 104/well) were seeded in culture six-well plates using William’s Medium E (Invitrogen, Carlsbad, CA, USA) and incubated at 37°C, 5% CO2. The medium was renewed every 3 days. Once a maximal confluence was reached (85 to 100%), culture medium was supplemented with EGF (90 ng/mL) during 1 week then with EGF and 2% dimethyl sulfoxide (DMSO) for another week to stimulate HepaRG cells differentiation. Biological triplicates were then treated with 0.5 mM, 1 mM, 2 mM O/P (2:1) (Sigma, St. Louis, MO, USA) in differentiation medium. Cells were washed with PBS and incubated with 100 nM Nile Red (Sigma) in the culture medium 15 min at 37°C. Cells were fixed in 4% formaldehyde, washed twice with PBS and mounted on a slide with a mounting medium containing DAPI for nuclear counterstaining. Cells were analyzed using a fluorescence microscope (Eclipse Ti, Nikon Instruments, Melville, NY, USA) and images were taken using the NIS-Elements software (NIS, Nikon Instruments).

### Beadarray analysis

Genomic DNA from all samples was treated with EZ DNA methylation-Gold kit (Zymo Research, Irvine, CA, USA), according to the manufacturer’s protocol to convert genomic DNA. The modified DNA (20 to 25 ng/μL) was stored at −20°C until use. The Illumina’s GoldenGate HCC methylation dataset has been previously reported [19,21]. Here, only the 153 CpG sites associated with imprinted genes have been filtered (corresponding to 59 imprinted genes). BRBArrayTools software (version 3.8 beta2) was used for further analysis, using the *M* values (Mi = log2(Betai/1 − Betai) as a transformation of the beta values, as recommended [44]. CpG sites showing minimal variation across the set of arrays were excluded from the analysis. Class comparison was performed with the BRBArrayTools software.

### Quantitative analysis of DNA methylation by pyrosequencing

The methylation status was confirmed using pyrosequencing assays, as previously described [21]. DNA amplifications, using specific biotinylated primers and specific PCR conditions, were carried out on all modified DNA samples (Additional file 4: Table S1). Of modified DNA, 20 to 25 ng were amplified in a total volume of 50 μL. Of PCR reaction, 10 μL were analyzed on agarose gel whereas the remaining 40 μL were used in pyrosequencing assay (Qiagen, Venlo, Netherlands) according to the manufacturer’s instructions.

### Quantitative RT-PCR

Total RNA was isolated using the TRIzol Reagent (Invitrogen) according to the manufacturer’s instructions. Reverse transcription reactions were performed using MMLV-RT (Invitrogen) and random hexamers, according to the manufacturer’s protocol. Primers and probes were designed using Universal Probe Library Assay Design Center (Roche, Basel, Switzerland). qRT-PCR was performed in triplicates of each condition, using FastStart TaqMan Probe Master (Roche) and a MX3000P real-time PCR system (Stratagene, San Diego, CA, USA).

#### The Cancer Genome Atlas data

HCC methylome data (idat files) and their related clinical data were obtained from TCGA Data Portal (https://tcga-data.nci.nih.gov/tcga/). Correlation analyses on DNA methylation and gene expression have been performed using MethHC, a database for human pan-cancer methylation and gene expression analyses [45]. Only complete datasets for DNA methylation and gene expression available for both tumors and adjacent matched non tumor samples were analyzed (47 cases). Data pre-processing and analysis was performed using R/Bioconductor packages. Data quality was assessed using boxplots for the distribution of methylated and unmethylated signals, and multidimensional scaling plots and unsupervised clustering were used to check for sample outliers. Cross-reactive probes, probes mapping to sex chromosomes, and probes overlapping with a single nucleotide polymorphism (SNP) with an allele frequency of at least 5% in the overall population were also removed, as previously described [46]. Type I and type II probe distributions were aligned using intra-array beta-mixture quantile normalization [47]. Logarithmically transformed methylation values [44] were interrogated for differential methylation between tumors and matched surrounding tissues in a paired linear regression [48]. DMPs were selected based on a threshold for the adjusted *P* value (FDR) of 0.05 and a difference in methylation between groups (delta-beta) of at least 40%. The bump hunting method was used to define DMRs using the recommended proximity-based criteria [49].

## Additional files

Additional file 1: Figure S1. Differential imprinted CpG methylation in HCC. Heatmap of all imprinted CpG sites (*n* = 153), with high methylation represented in red and low methylation in blue. The unsupervised clustering is able to discriminate HCC tumors from adjacent tissues, as shown in the lower bar annotation. Additional file 2: Figure S2. TCGA data analysis. A. Heatmap of all differentially methylated positions distinguishing HCC tissues from their matched surrounding tissues (*n* = 1,328), with high methylation represented in red and low methylation in blue. B. average methylation levels for a selection of imprinted genes was plotted using MethHC (as described in Methods). Tumors are shown in red, and non-tumor tissues are shown in green. Additional file 3: Figure S3.
*In vitro* steatosis model. Lipid accumulation in cells treated with Oleate/Palmitate (O/P). Lipid vacuole accumulation was assessed after 48 h in control cells (A) and cells treated with 0.5 mM (B), 1 mM (C), 2 mM (D) O/P (200×). Lipids were stained with Nile red (red) and nuclei were counterstained with DAPI (blue) (upper panel). DNA from these conditions was used to assess DNA methylation by pyrosequencing of selected imprinted regions (lower panels). (*) indicates *P* value <0.05. Additional file 4: Table S1. Pyrosequencing assays. List of primers (forward, reverse, and sequencing) used for bisulfite-pyrosequencing assays of imprinted loci. ICR = imprinting control region.

## Abbreviations

DMR: differentially methylated region
FDR: false discovery rate
HBV: hepatitis B virus
HCC: hepatocellular carcinoma
ICR: imprinting control region
