# Supplementary figures and images for: Aberrant DNA methylation of imprinted loci in hepatocellular carcinoma and after in vitro exposure to common risk factors
Source: Clin Epigenetics. 2015 Feb 27;7(1):15. doi: 10.1186/s13148-015-0053-9 (PMC4353474; doi:10.1186/s13148-015-0053-9)

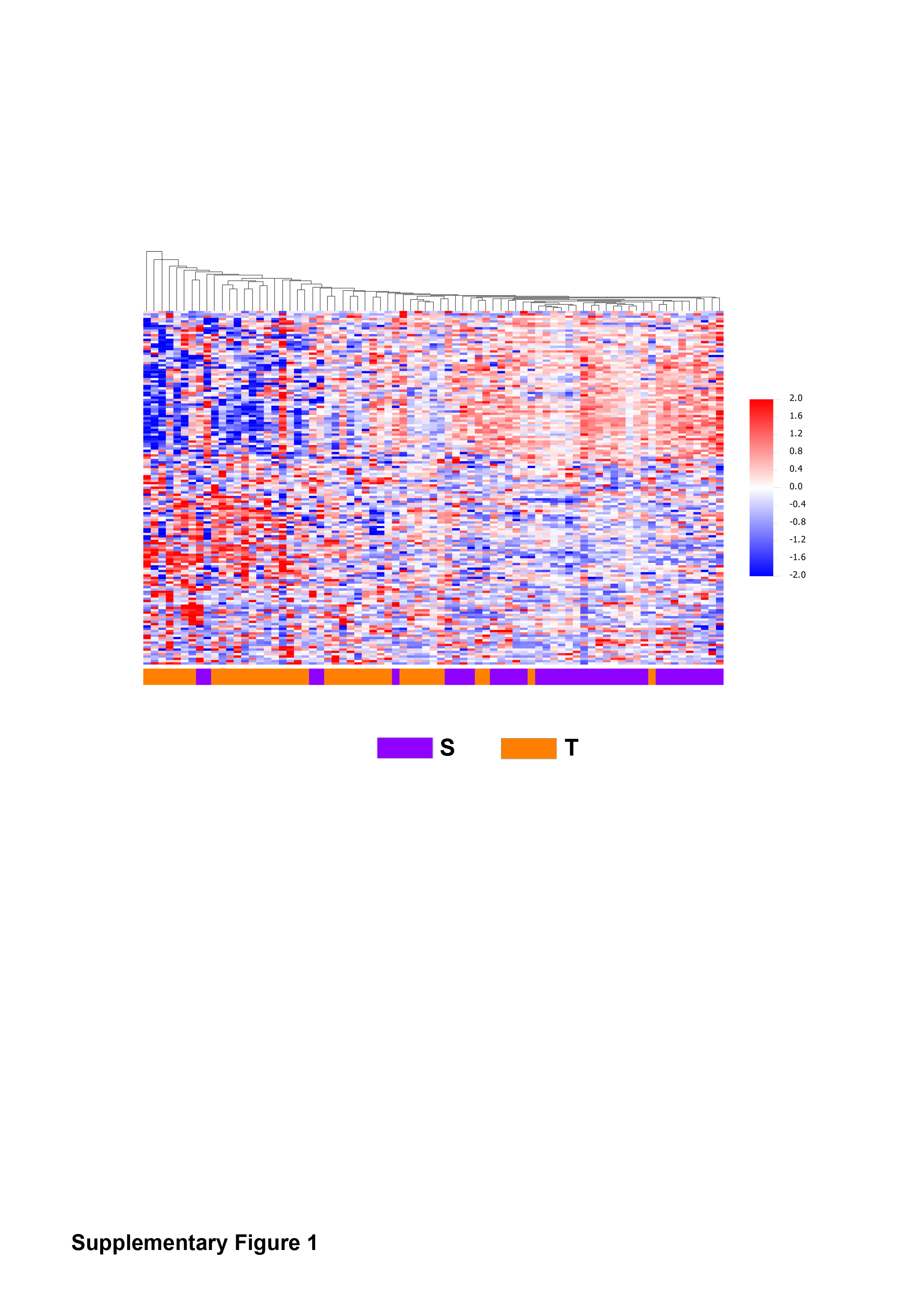

Supplement: Additional file 1: Figure S1. — Differential imprinted CpG methylation in HCC. Heatmap of all imprinted CpG sites (n = 153), with high methylation represented in red and low methylation in blue. The unsupervised clustering is able to discriminate HCC tumors from adjacent tissues, as shown in the lower bar annotation. [file 13148_2015_53_MOESM1_ESM.tiff]

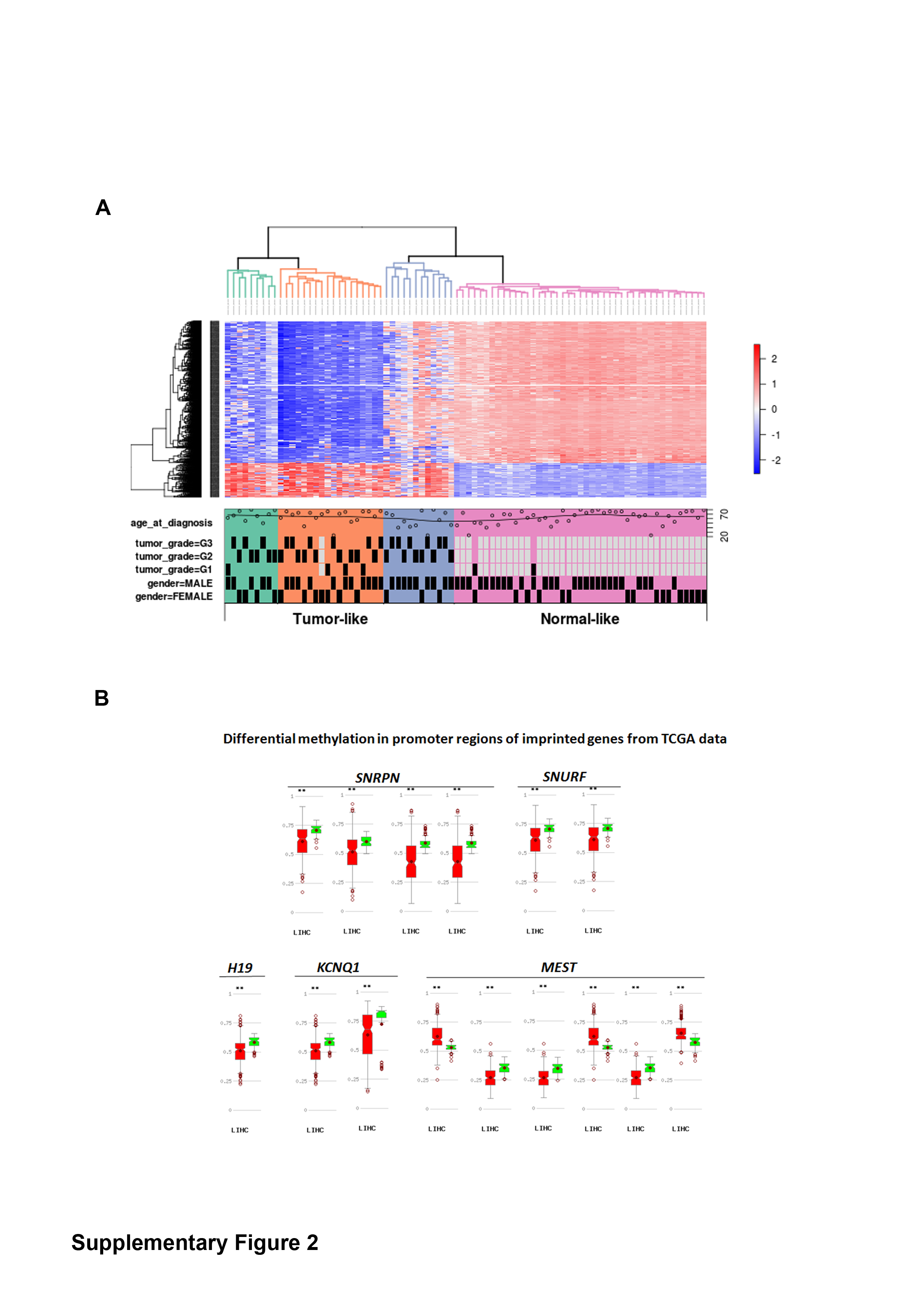

Supplement: Additional file 2: Figure S2. — TCGA data analysis. A. Heatmap of all differentially methylated positions distinguishing HCC tissues from their matched surrounding tissues (n = 1,328), with high methylation represented in red and low methylation in blue. B. average methylation levels for a selection of imprinted genes was plotted using MethHC (as described in Methods). Tumors are shown in red, and non-tumor tissues are shown in green. [file 13148_2015_53_MOESM2_ESM.tiff]

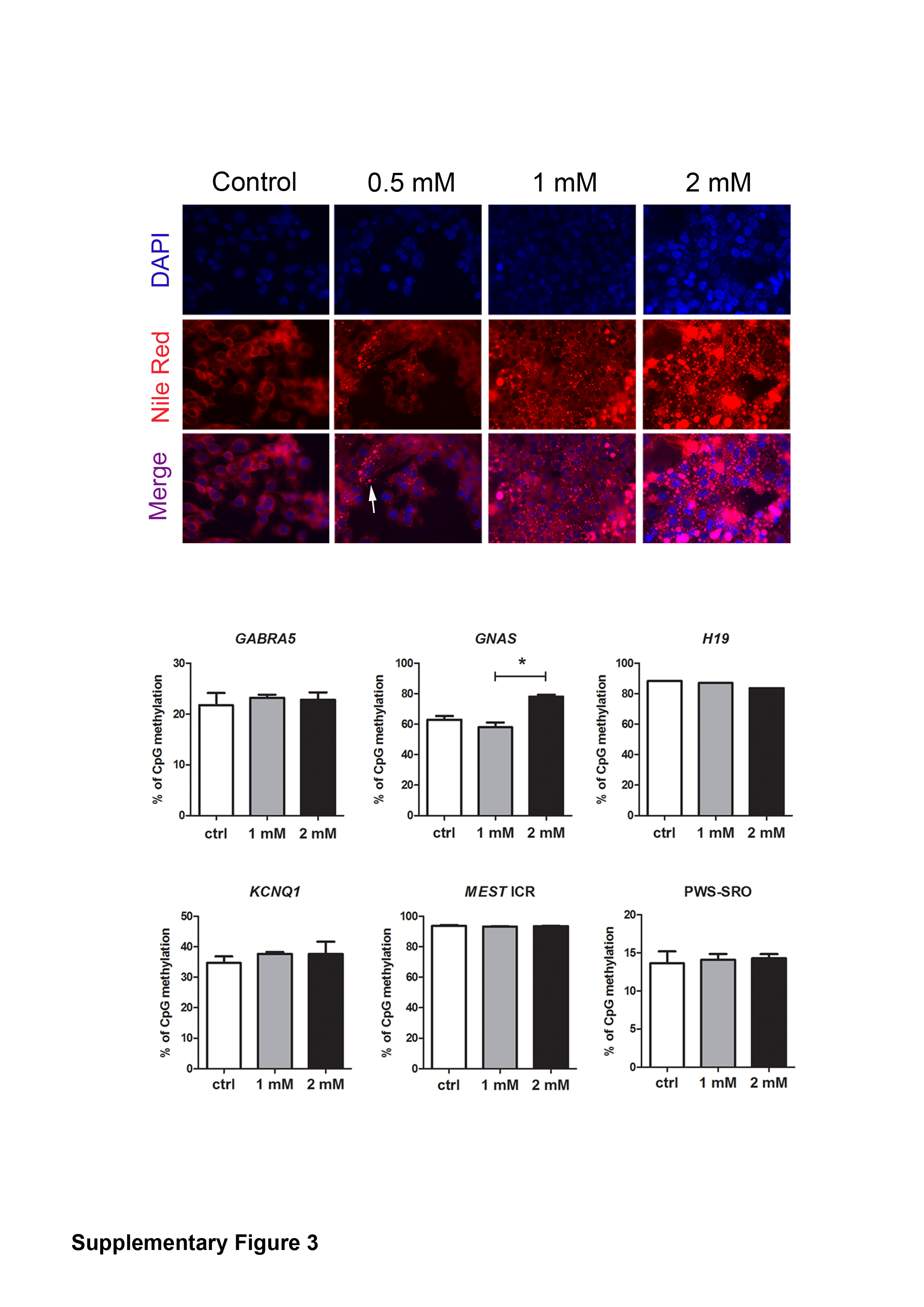

Supplement: Additional file 3: Figure S3. — In vitro steatosis model. Lipid accumulation in cells treated with Oleate/Palmitate (O/P). Lipid vacuole accumulation was assessed after 48 h in control cells (A) and cells treated with 0.5 mM (B), 1 mM (C), 2 mM (D) O/P (200×). Lipids were stained with Nile red (red) and nuclei were counterstained with DAPI (blue) (upper panel). DNA from these conditions was used to assess DNA methylation by pyrosequencing of selected imprinted regions (lower panels). (*) indicates P value <0.05. [file 13148_2015_53_MOESM3_ESM.tiff]
